# Dynamic accelerated stress test and coupled on-line analysis program to elucidate aging processes in proton exchange membrane fuel cells

**DOI:** 10.1038/s41598-024-54258-8

**Published:** 2024-02-18

**Authors:** Lena Birkner, Michael Foreta, Ali Rinaldi, Anton Orekhov, Marc-Georg Willinger, Maik Eichelbaum

**Affiliations:** 1 Nuremberg Institute of Technology, Institute for Applied Hydrogen Research, Electro- and Thermochemical Energy Systems (H2OHM), 90489 Nuremberg, Germany; 2 MAN Truck & Bus SE, Material Technology and Applied Chemistry (EOMC), 90441 Nuremberg, Germany; 3 Technical University of Munich, Chair of Electron Microscopy, 85748 Garching, Germany

**Keywords:** Chemistry, Energy science and technology

## Abstract

The application of hydrogen proton exchange membrane fuel cells (PEMFC) in greenhouse gas emission free heavy-duty vehicles requires extremely durable PEMFC components with service lives in the range of 30,000 h. Hence suitable test and analysis methods are required that reflect realistic operation scenarios, but significantly accelerate aging. For this purpose, a dynamic accelerated stress test was developed, which is coupled with a comprehensive in-depth in-situ and ex-situ analysis program to determine the aging processes of a PEMFC membrane electrode assembly (MEA). The test comprehends dynamic cycling between low, moderate and high load, different temperature and humidity conditions as well as recovery sequences to distinguish between reversible and irreversible failure modes. All phases of the PEMFC system (i.e. solid, liquid and gaseous) are monitored on-line during aging by sophisticated electrochemical, mass spectrometric and ion chromatographic analytical methods. The structural and elemental composition of the MEA before and after the aging program (post-mortem) are investigated by X-ray fluorescence, scanning and transmission electron microscopy. This program was able to age a commercial PEMFC to end-of-life in 1000 h, while providing an accurate picture of the aging processes involved.

## Introduction

Hydrogen and hydrogen-based fuel cell technologies have been discussed and researched for decades as a necessary component of a defossilized energy system^1,2^. The dramatic escalation of the climate change and the current raw material and energy crisis provide strong arguments for a fast implementation of a hydrogen economy. The defossilization of the entire energy system makes energy vectors with a high energy density necessary to store, transport and convert energy when and where it is needed^1^. The advantage of hydrogen is its versatility to operate across the transport, heat, industry and electricity sectors, which account for about two-thirds of global ${\text{CO}}_2$ emissions^2^. European, U.S. and global hydrogen roadmaps indicate, that up to 25 % of the total energy demands could be met by hydrogen by 2050, of which 30 % is expected to be used for the transportation sector^3^. Fuel cells are essential in this scenario, since they can convert green hydrogen produced by water electrolysis using renewable energy resources into electricity and heat. At the moment, an increasing interest in fuel cell technologies can be observed^2^. Although material and production costs have to be reduced further by production up-scaling and material innovations^4^, the increasing relevance of fuel cells is particularly evident in the mobility sector due to the shift in research and technology focus from passenger cars to heavy-duty vehicles (HDV)^3^. In the case of HDVs, PEMFCs could ideally exploit their advantages. Fuel cells can be scaled in terms of both power and energy, with an increase in the size of the hydrogen tank and fuel cell stack accompanied by a much smaller increase in weight than with battery-only drives. Moreover, PEMFC-based powertrains are much more efficient than internal combustion enegines (ICE) and do not emit nitrogen oxides while promising a similar driving range and refueling time.

However, the successful commercialization of fuel cells in the transport sector is still hampered by fundamental challenges^2^. Compared to light duty vehicle (LDV) applications, operational expenditures play a greater role than capital expenditures for HDVs due to their much larger daily and lifetime mileages. In addition, HDVs are more often operated near maximum power than LDVs. The previous research and technology focus was mainly on reducing fuel cell costs for LDV applications. Now, for HDV applications with their long lifetimes, the end-of-life performance and thus the fuel cell durability is of much greater importance. The U.S. Department of Energy (DoE) has set an intermediate fuel cell lifetime target for HDV applications of 25,000 h by 2030^3,5,6^. The final target has been set at 30,000 h to achieve similar user acceptance as diesel-powered HDVs. Leading fuel cell manufacturers currently (2023) quote lifetimes of up to 20,000 h for their products. Typical failure modes restricting the durability of fuel cells comprehend, e.g., reduction of the platinum electrochemically active surface area, catalyst carbon support corrosion, degeneration of the polymer electrolyte membrane and mass transport limitations due to a loss of hydrophobicity of the gas diffusion and microporous layer (GDL/MPL)^7–15^. Due to the long durability of PEMFCs in the range of tens of thousand hours needed for HDV applications, aging and lifetime tests can be very time consuming and expensive. Therefore, accelerated stress tests (AST) are needed to predict the service life under laboratory conditions and on much shorter time scales^16^. In the last years, various ASTs have been developed for PEMFCs^17–21^. DoE and European Commission, among others, suggested standardized ASTs that can account for the specific aging of the entire PEMFC or of selected fuel cell components^22,23^. E.g., corrosion of the high-surface area carbon support can be initiated by applying high electrode potentials, start/stop cycles and high temperatures. The membrane can be mechanically aged by cycling the gas humidity and temperature, while the chemical stability of the entire MEA is stressed by running the fuel cell under open-circuit voltage (OCV) conditions. Platinum catalyst particle growth and migration can be accelerated under high electrode potentials and by load cycling. For the GDL/MPL, freeze-thaw cycling or high temperature treatments could be identified as most important stress factors. However, specific aging conditions can affect many different parts of the fuel cell, and aging mechanisms are usually coupled^19^. For example, AST-induced migration of the platinum catalyst into the PEM can catalyze the formation of radicals that can destroy the membrane^24^. Increasing hydrogen and oxygen gas permeability of the degraded PEM accelerates the formation of highly oxidative radicals that can attack and degrade the carbon support^25^, etc. Moreover, under realistic conditions such as in a typical driving cycle, various stressors will simultaneously challenge the stability of the fuel cell. In addition, long-term aging based on singular stressors or of singular PEMFC components can overestimate the aging or even introduce new degradation processes. Studies have shown that typical degradation modes such as platinum oxidation, platinum nanoparticle migration or carbon support corrosion can be reversed to a certain extent with suitable recovery procedures^26^. Such reversible processes could also be relevant under normal operation conditions. Determining the service life under one-sided extreme conditions without taking reversible processes into account can therefore lead to incorrect conclusions. As a consequence, it is desirable to develop AST programs that can simulate stressing conditions as realistic as possible without adding any new failure modes.

The aim of our work is hence to develop an advanced AST program that fulfills the following prerequisites: i) It reproduces realistic conditions and aging effects observed during typical driving cycles in HDV applications. ii) It comprehends the aging of all important MEA components, i.e. catalyst layer (CL), carbon support, membrane and GDL/MPL. iii) It comprimates significantly the total fuel cell cell lifetime. iv) It allows interpretations of the reversibility and irreversibility of the involved deactivation steps. v) It enables an efficient on-line monitoring of the aging processes. We have deliberately avoided extreme conditions such as freezing temperatures, temperatures above 100 ^∘^C, additional introduced impurities or potentials above the OCV in order to initially exclude these special aging influences. Instead, the AST includes a dynamic load cycle reflecting various typical driving conditions. On this basis, we have programmed a loop of repetitive AST sequences representing an alternation of moderate and demanding loads, humiditities and temperatures. After each sequence, a defined analysis program is performed with on-line monitoring of all phases of the PEMFC system (i.e. solid, liquid, gas). At the very beginning and at the end of the PEMFC lifetime, structural and elemental analyses are also performed on the MEA. Through this workflow, we obtained a very detailed picture of the relationships between the stressors and their respective component-specific aging effects, including insights into reversible and irreversible deactivation mechanisms.

## Results

### Materials choice

Since this study comprehends the introduction of a new testing protocol for fuel cells, we have chosen the MEA as described in the “Methods” section for a first proof-of-principle test out of the following reasons: 1) This MEA is commercially available in large numbers and a broad variety of sizes (i.e. it is accessible to many research groups and can be (or was already) used as benchmark or “generic” fuel cell to compare different test protocols on different test benches with the same kind of materials). 2) The performance and aging behavior of this or similar kind of MEA and components is already well known and described in the literature^27–32^. This allowed us to test our applied analytical methods for their ability to diagnose typical failure modes that are expected for this kind of MEA. And 3) the MEA was selected because the measured electrochemical and microscopic data can be published without any legal restrictions and can hence be provided in all detail to the scientific community. E.g., the platinum loading of 0.5 mg/cm^2^ on each electrode side was selected to avoid a too early failure due to the loss of catalytically active surface. This is also justifiable since durability and efficiency (and hence lower operational expenditures) are for long lifetime HDV applications more important than capital expenditures (and hence very low platinum loadings as in LDV applications)^3^. The membrane NR-212 was chosen due to its moderate thickness of 50.8 $\upmu$m balancing transport limitations and mechanical stability. As for the carbon support and the GDL/MPL standard materials were used. We deliberately avoided the introduction of more sophisticated materials and modifications since we wanted to set a first benchmark with this well described MEA, whereas the aging processes possibly associated with new materials are less or not at all described or even well understood. However, we already tested a commercial MEA that was specifically designed for the usage in demanding HDV applications and provide first results in a later chapter of this manuscript. Hence we could prove that our test protocol is also able to age such kind of highly sophisticated materials in a reasonable time frame.

### Accelerated stress test (AST)

**Figure 1:**
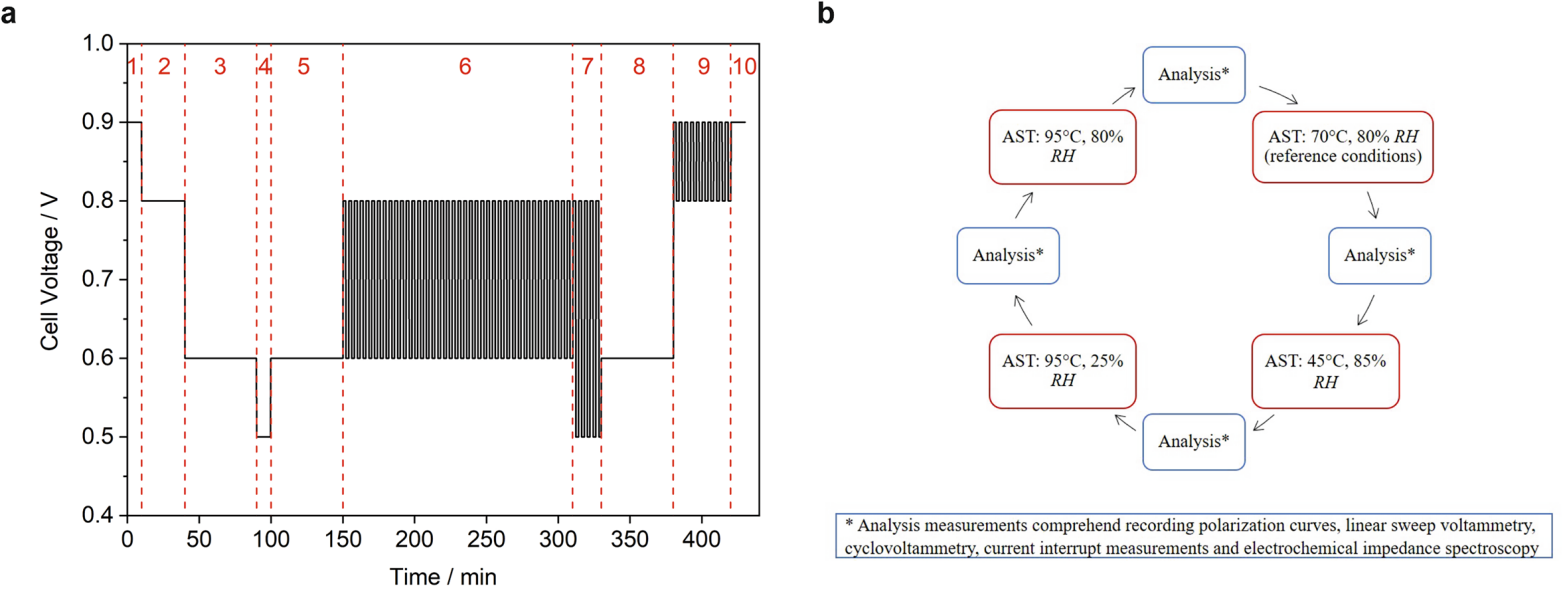
(**a**) Single (iR drop compensated) voltage cycle applied as accelerated stress test (AST) to the investigated fuel cell MEA. Note that the applied maximum voltage has been adjusted to the achievable OCV. (**b**) Complete AST/analysis program loop including AST voltage cycles at different temperatures and relative gas humidities (*RH*) as well as analysis sections under reference conditions after each AST cycle.

In order to tailor the AST to typical HDV operating conditions, an iR drop compensated voltage cycle developed for mobile applications^33^ was applied at various fuel cell temperatures and relative gas humidities to cause accelerated but realistic degradation of a single PEMFC MEA. The cathode was purged with humidified filtered air and the anode with humidified hydrogen, both at ambient pressure. The voltage cycles were repeated at four different temperature and gas humidity conditions in order to simulate medium temperature/high humidity (= reference conditions), low temperature/high humidity, high temperature/low humidity and high temperature/high humidity conditions. Temperature and humidity cycling are also known as in-situ stressors for membrane, GDL/MPL and platinum CL^34^. After each single voltage cycle an electrochemical analysis cycle was run under reference conditions. Only during both the cyclovoltammetry and linear sweep voltage measurement, the cathode was purged with nitrogen. This had the positive side-effect that possibly reversible aging processes were reversed and excess water was removed as suggested by Dhanushkodi et al.^35,36^ Moreover, we introduced a dry sequence with a turned-off gas humidifier between AST cycle 4 and 8 (i.e., between 14 and 50 h aging time). A single applied voltage cycle is depicted in Fig. 1a and the complete AST and analysis program loop is shown in Fig. 1b. This program was repeated until a total fuel cell AST aging time of 1000 h was achieved. (Note that the analysis time was not counted as aging time.) After this time, several end of life parameters were reached and the continuation of fuel cell operation would have become a safety issue.

The AST voltage cycle can be divided into several sections that mark different characteristic operating points of the fuel cell and are thus intended to simulate a comprehensive driving cycle in a PEMFC-operated HDV. The different sections are based on typical HDV drive cycles such as the HDV drive cycle for a Class 8 truck defined by the California Air Resource Board^3^. Special emphasis was also placed on the integration of typical stressors described in the literature for the accelerated degradation of the specific PEMFC MEA components^34^. Sections 1 and 10 represent phases under OCV. The OCV state, i.e. a phase without power consumption, is usually applied in a vehicle as part of the shutdown (SD) or startup (SU) procedure^37,38^. OCV conditions are also applied in the DoE membrane chemical AST^34^. It should be noted that there are various SD and SU procedures possible in order to avoid a potentially destructive long-term OCV state, e.g. by purging the anode with air or by a controlled consumption of residual hydrogen and oxygen at the electrodes at low load^37^. These special SD/SU processes were not simulated in our AST program, as this can add new aging effects (e.g. due to the formation of hydrogen/air fronts)^38^. This depends on the particular operating conditions chosen by the manufacturer, but could be added to the AST if known. Section 2 includes a constant phase at 0.8 V, which could represent idling or low load conditions. Idling, i.e. operation at low power consumption, is required to operate the engine in certain situations even though the vehicle is not moving. For example, hydraulic or cooling units are often required during loading processes, which need power that the engine must provide. In addition, the engine is usually not switched off during breaks in very warm or cold temperatures in order to keep the heating or air condition on. Moreover, a minimum idle power has been recommended for HDV fuel cell applications to limit the peak cathode potential below the OCV in order to avoid cathode catalyst degradation^3^. The value of 0.8 V has also been chosen since it is a common operation point of the PEMFC Class 8 truck drive cycle simulated by the Argonne National Laboratory^3^ and it is part of the dynamic stress test (DST) developed by the DoE and US Fuel Cell Council^39^. Section 9 represents a rapid change between idling/low load and OCV conditions, simulating the phase between SD or SU and motor switch-on/off for the former reasons. In sections 3, 5 and 8, a voltage of 0.6 V is maintained to simulate a constant intermediate load. In this phase, driving under moderate conditions (i.e. under moderate speed, loading, acceleration etc.) is simulated. In section 4, a voltage of 0.5 V is maintained, which corresponds to overload operation. Note that operation of PEMFCs at higher cell voltages allows higher efficiencies and operation at lower temperatures, but also causes a lower power density and hence larger stack size at rated power^3^. At 0.5 V the vehicle is driving under extreme (and inefficient) conditions (with the generation of much parasitic waste heat), e.g. under heavy acceleration, uphill, fully loaded, in difficult environments (muddy or snow-covered paths etc.) and/or at top speed. We added this section in order to stress the PEMFC close to the mass transport limitation range. Sections 6 and 7 reflect highly dynamic cycles, i.e. changes between 0.6 and 0.8 V (change between intermediate load and idling/low load, e.g. when the vehicle comes to a standstill and accelerates moderately again) and 0.5 and 0.8 V (change between overload and idling/low load, e.g. when the vehicle accelerates very strongly or drives uphill from a standstill). The cycling between low and high potentials also resembles the DoE square wave potential platinum catalyst durability test^34^.

Electroanalytical characterization associated with the analysis cycles in the program includes recording of polarization curves, linear sweep voltammetry (for the determination of hydrogen crossover currents and internal membrane resistance), cyclic voltammetry (for the calculation of the electrochemical surface area), current interruption (for the measurement of the ohmic resistance) as well as electrochemical impedance spectroscopy. To ensure comparability, all electroanalytical measurements were performed at defined (reference) conditions comprising a temperature of 70 ^∘^C and a relative humidity of 80 %. In addition, the cathode exhaust gas from the PEMFC was continuously analyzed on-line by mass spectrometry. Moreover, the fuel cell water from the cathode exhaust was condensed and collected in stainless steel vessels and regularly analyzed for platinum by voltammetry and for fluoride by ion chromatography.

### Electrochemical characterization

#### Polarization measurements

**Figure 2:**
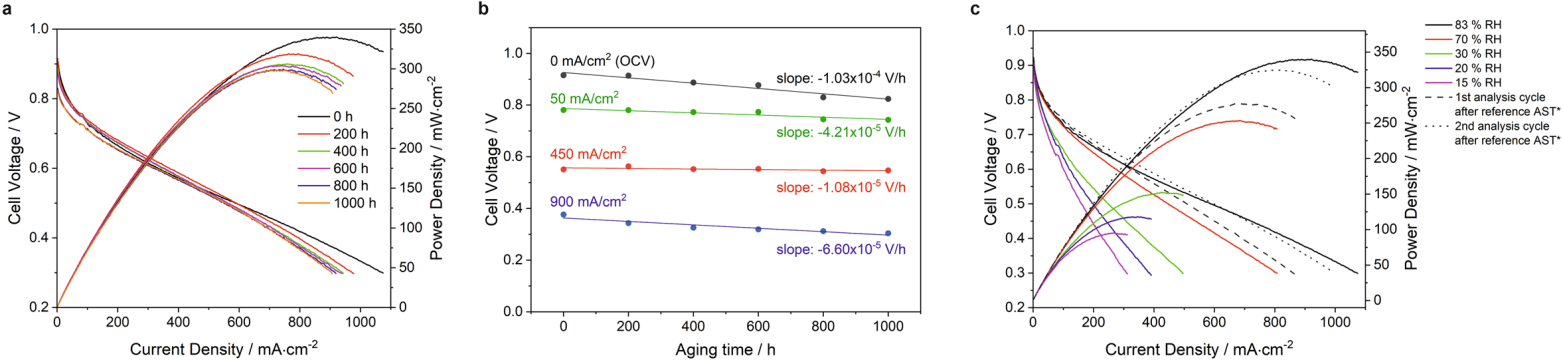
(**a**) Current and power density diagrams recorded after different times of the applied AST program. All curves were measured in the analysis cycle directly after the AST run under reference conditions. (**b**) Cell voltages at indicated current densities, measured by recording polarization curves during the analysis cycles directly after the AST run under reference conditions. The lines represent linear regressions of data points which obtained the slopes given in the diagram. (**c**) Current and power density diagrams recorded between the 4th and 8th AST cycle with turned-off gas humidifier. All curves were measured during the analysis cycle after the AST run under reference conditions. *Dashed and dotted curves show the behavior of the PEMFC after turning-on the humidifier again.

In order to characterize the fuel cell upon AST aging, polarization curves were recorded in every analysis step of the AST program. The obtained current and power density diagrams measured in aging time intervals of 200 h are summarized in Fig. 2a. After 1000 h aging, the peak power density decreased from 339.5 to 298.4 mW/cm^2^, i.e. by 12.1 %. The trend of the cell voltages was deduced by plotting the voltages measured at current densities of 0, 50, 450 and 900 mA/cm^2^, respectively, versus aging time (Fig. 2b). While the 0 mA/cm^2^ data represent the OCV, the other values are characteristic for the activation, ohmic and mass transport section of the polarization curve, respectively. As a result, the largest total decrease was observed for the OCV. The cell voltage dropped from 0.915 to 0.824 V, i.e. by 10.0 %. A smaller absolute and relative change from 0.781 to 0.743 V (4.8 %) was observed in the activation region. The smallest both relative and absolute voltage decline was monitored in the ohmic region with a decrease from 0.551 to 0.547 V (0.73 %). The largest relative change of 19.2 % could be measured in the mass transport region with a voltage drop from 0.376 to 0.304 V. Moreover, all the voltage changes are rather linear with time in all four current density regions as shown by the results of the linear regression depicted in Fig. 2b. The slope of the linear regressions gives the respective voltage changes per hour as stated in Fig. 2b.

In addition to the regular AST program, the humidifier was turned off between the 4th and 8th AST cycle inducing a continuous decrease of the humidity of the input gases to a minimum value of 15 %*RH*. During this interval, the peak power density decreased from the maximum of 339.5 to a value of only 94.2 mW/cm^2^ (relative decrease by 72 %). After turning on the humidifier again, the power density started to recover. However it took two complete AST/analysis program loops to obtain a maximum value of 324.7 mW/cm^2^, which was still 4.4 % below the original peak power density (Fig. 2c).

**Figure 3:**
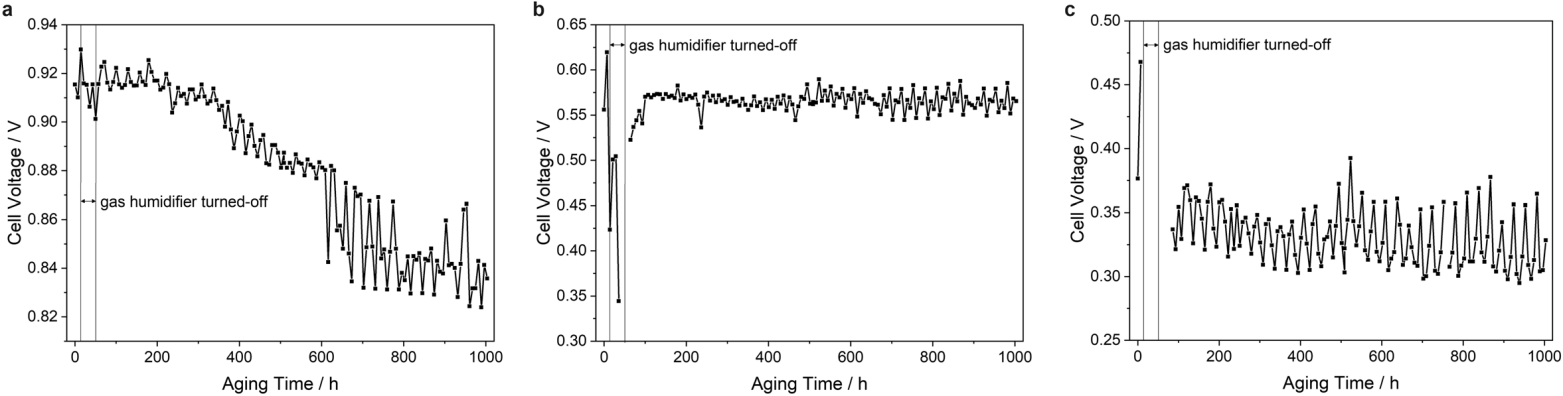
Cell voltages recorded at (**a**) OCV, (**b**) 450 mA/cm^2^ and (**c**) 900 mA/cm^2^ during AST aging recorded by measuring polarization curves in the respective analysis sections of the AST program.

The aging during the AST program including the low humidity phase was investigated in more detail by plotting all measured cell voltages recorded during all analysis cycles at OCV, 450 and 900 mA/cm^2^, respectively, versus aging time (Fig. 3). The oscillation of the cell voltages is a result of the polarization measurements following the different AST cycles at different temperatures and humidities. Even though the polarization curves were always recorded under the same conditions, the previous treatment is obviously still observable in the respective data points. In general, in the first half of the aging program the highest voltages were usually observed in the analysis step following the 95 ^∘^C/80 % *RH* treatment, while the lowest voltages were measured after the 45 ^∘^C/85 % *RH* cycle, in the ohmic region also sometimes after the 95 ^∘^C/25 % *RH* conditions, though the differences in this region were not very high. An exception is the mass transport region, where in this first phase the maximum cell voltage was in most cycles obtained after the 45 ^∘^C/85 % *RH* and the lowest cell voltage after the 95 ^∘^C/80 % *RH* treatment. However, this changes fundamentally in the second half of the aging protocol, when generally the maximum cell voltage was reached after the 95 ^∘^C/25 % *RH* treatment and the lowest cell voltage was observed following the 95 ^∘^C/80 % RH treatment for all current densities.

In detail, the OCV minimum dropped by 0.99 %, i.e. from 0.910 before to 0.901 V during the low humidity treatment, but quickly recovered to 0.913 V under “normal” humidity conditions, indicating that this deactivation was completely reversible (Fig. 3a). Interestingly, the OCV remained rather constant until about 200 h of aging. At longer aging times the OCV declined continuously. The values oscillated between the different meaurement points by about 15 mV. This situation changed after 600 h of aging. From then on, neighbouring data points differed by more than 40 mV.

The behavior of the voltage was very different in the ohmic range (Fig. 3b). Here, the voltage minimum dropped from 0.556 to less than 0.344 V (relative decline > 38 %) under low humidities. Lower values could not be measured since the polarization measurement stopped at voltages below 0.3 V. After turning on the humidifier again, it took about 50 h until the voltages recovered to the original values. However, from then on nearly no decline was observed until the end of the experiment. Only the oscillations between highest and lowest voltage values started to increase after about 460 aging hours. While the difference of neighbouring data points was before that time typically less than 8 mV, the fluctuations increased to more than 30 mV at the end of the AST.

The voltages in the mass transport region shown in Fig. 3c fell below 0.3 V under the low humidity conditions and were therefore not recorded. Before this low humidity phase, the voltage increased from 0.376 up to 0.468 V indicating an initial acitivation of the fuel cell during the first hours. This maximum was never reached again after turning on the humidifiers, which gives evidence that the mass transport was irreversibly deteriorated. The highest cell voltage after turning on the humidifier again was measured with 0.393 V after 523 h. The oscillations between neighboring points were over the whole aging treatment higher than in all the other polarization regions with typical values of about 35 mV until 450 h of aging. At longer aging times, the differences between minimum and maximum voltage increased to more than 60 mV.

#### Linear sweep voltammetry (LSV)

**Figure 4:**
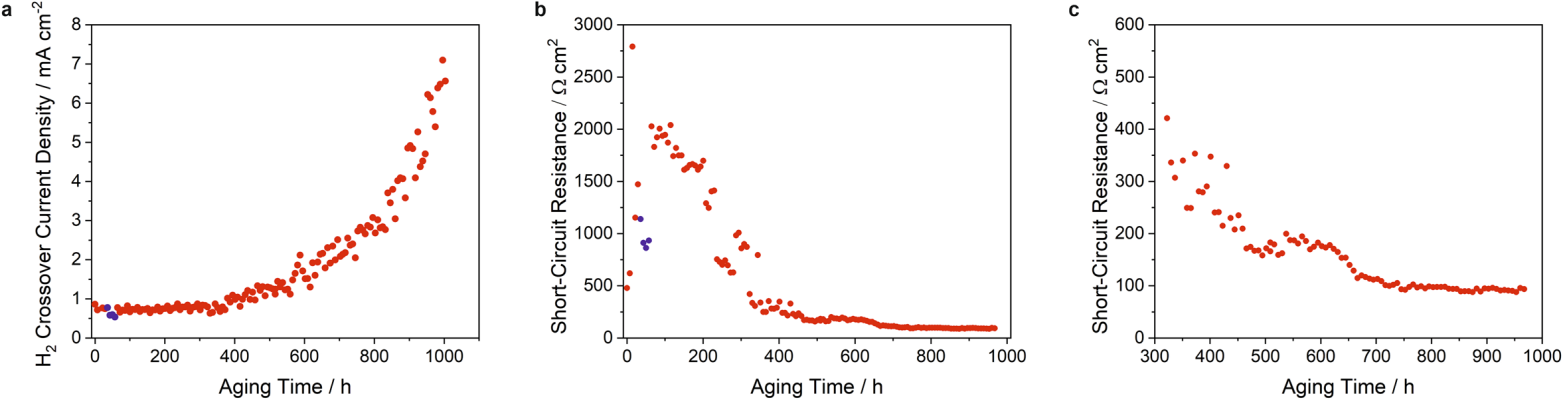
(**a**) H_2_ crossover current density and (**b**) short-circuit resistance as measured by linear sweep voltammetry. Blue data points represent the measurement with turned-off humidifier. (**c**) Enlarged section of the short-circuit resistance between 300 and 1000 h of aging.

LSV was applied to determine the hydrogen crossover current density and the electrical short-circuit resistance as characteristic indicators for the state of health of the membrane. During the first 370 h of aging, the crossover current density remained permanently below 1 mA/cm^2^ (Fig. 4a). During the phase with turned-off humidifier the values dropped to a minimum of 0.53 mA/cm^2^ (blue data points in Fig. 4a), but recovered immediately to current densities of about 0.7 mA/cm^2^ after turning on the humidifier. Starting at about 400 h, the crossover current density increased nearly linearly to more than 3 mA/cm^2^ until 800 h of aging. Afterwards, an exponential rise was observed. Note, that the potentiostat used in this program had a measurement limit of 8.57 mA/cm^2^. This value was exceeded after 970 h.

Figures 4b and c show the calculated short-circuit resistance. At the beginning of the AST, the resistance increased to values of more than 2,700 $\Omega$ cm^2^ indicating an activation of the membrane. The low humidity phase stopped this and induced a drop of the values to about 860 $\Omega$ cm^2^ (blue data points in Fig. 4b). After increasing the humidity again, the resistance immediately recovered achieving more than 2,000 $\Omega$ cm^2^. However, after 120 h of aging the resistance started to fall again reaching values of about 170 $\Omega \,$cm^2^ after 465 h. The resistance stayed rather constant until 620 h and then fell again to about 90 $\Omega$ cm^2^. After 970 h, no values could be deduced due to the increase of the current density above the measurement limit of the potentiostat.

#### Cyclovoltammetry (CV)

**Figure 5:**
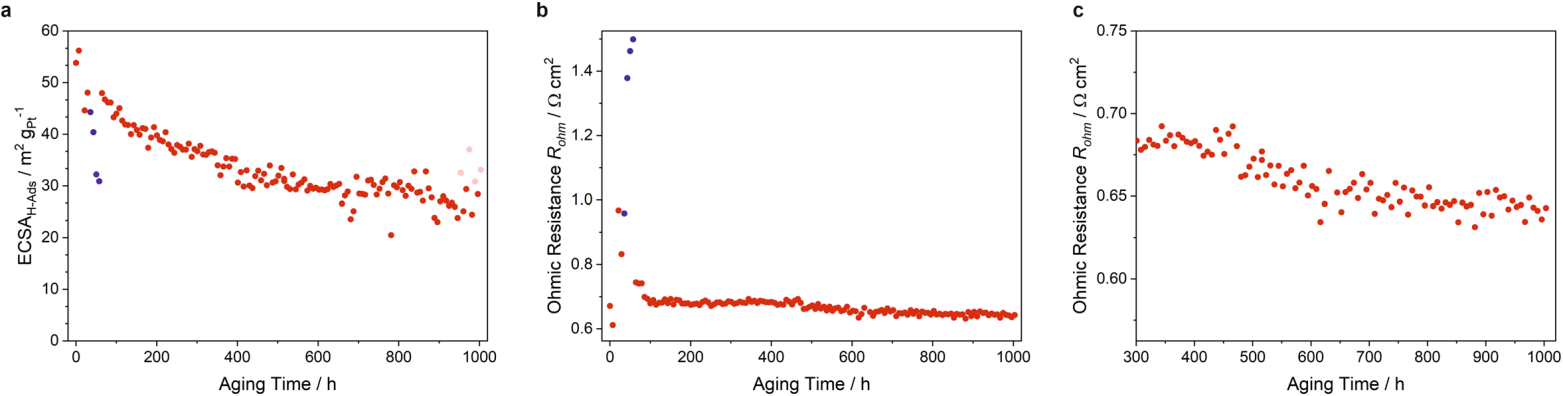
(**a**) Electrochemical surface area (ECSA) of the platinum catalyst as measured by cyclovoltammetry and (**b**) ohmic resistance as measured by current interruption. (**c**) Enlarged section of the ohmic resistance between 300 and 1000 h of aging. Blue data points represent the measurement with turned-off humidifier. The light red points in (**a**) were deduced from cyclovoltammograms with very small hydrogen desorption peaks causing a high error of peak integration.

The electrochemical surface area (ECSA) of the platinum catalyst as measured by CV is depicted in Fig. 5a. The surface was steadily decreasing throughout the whole aging experiment with the largest changes at the beginning. A sharp drop from 48 to 31 m^2^/g_Pt_ was observed during the turned-off humidifier (blue data points in Fig. 4b). With turned-on humidifier the ECSA immediately recovered and increased to 48 m^2^/g_Pt_. Subsequently, the surface area decreased to about 30 m^2^/g_Pt_ after 580 h of aging. From then on the values started to strongly fluctuate around this value, which is likely due to larger errors of the ECSA estimation by integrating small hydrogen desorption peaks in the CV. After 880 h, there seems to be a trend to even smaller surface areas.

#### Current interruption (CI)

The ohmic resistance of the MEA was measured by the CI technique. The resistance increased initially from 0.68 to 0.98 $\Omega$ cm^2^, dropped to 0.83 and increased again to a total maximum of 1.5 $\Omega$ cm^2^ with turned-off humidifier (Fig. 5b). Afterwards, the resistance fell sharply to 0.75 $\Omega$ cm^2^. After about 115 h, the values ramained rather stable at about 0.68 $\Omega$ cm^2^. After 480 h, the resistance started to decrease again reaching values of around 0.63 $\Omega$ cm^2^ at the end of the experiment (Fig. 5c).

#### Electrochemical impedance spectroscopy (EIS)

**Figure 6:**
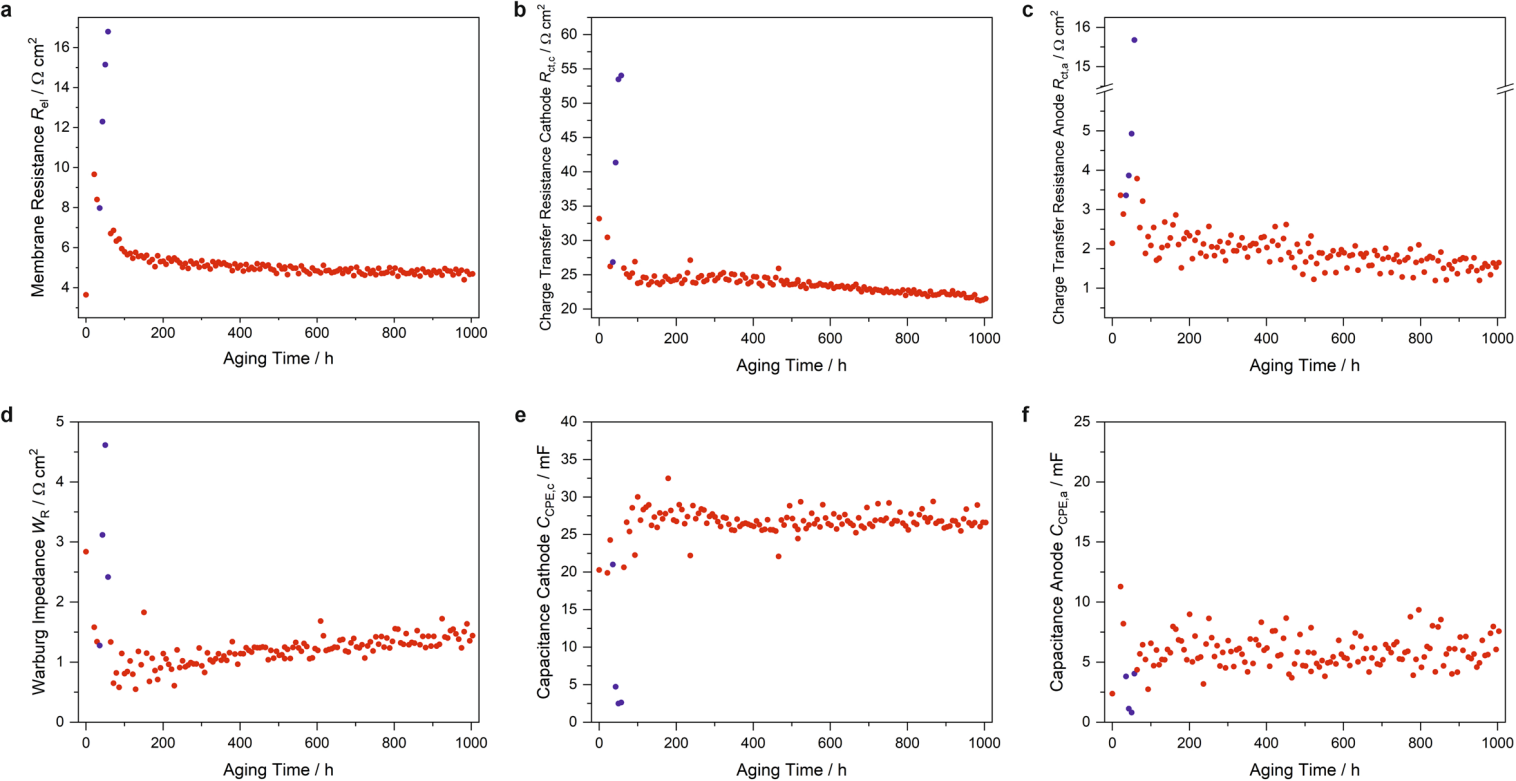
Parameters from EIS measurements during AST aging deduced via equivalent circuit fits. (**a**) Membrane resistance, (**b**) charge transfer resistance of the cathode, (**c**) charge transfer resistence of the anode, (**d**) Warburg impedance, (**e**) constant phase element (CPE) capacitance of the cathode and (**f**) CPE capacitance of the anode. Blue data points represent measurements with turned-off humidifier.

EIS data were recored in each analysis section of the AST program. By applying equivalent circuit fits to the spectra, values for charge transfer resistances of the cathode ($R_{ct,c}$) and anode ($R_{ct,a}$), for electrochemical double layer capacitances of the cathode ($C_{CPE,c}$) and anode ($C_{CPE,a}$), for the membrane resistance ($R_{el}$) and for the Warburg impedance ($W_R$) were deduced during each aging step. The obtained EIS parameters in dependence on aging time are summarized in Fig. 6, the exponents of the CPE capacitances are shown in Fig. S1.The EIS resistance and capacitance values tentatively assigned to the anode are not interpreted due to their minor contribution to the complex impedance data and the hence unsecure assignment to a single electrode side.

Obviously, all resistances including the Warburg impedance increased significantly with turned-off humidifier. The membrane resistance initially increased under normal humidity conditions from 3.8 to 9.5 $\Omega$ cm^2^ and peaked in the low humidity regime to a maximum of 16.8 $\Omega$ cm^2^. After turning on the humidifier, the resistance fell very fast to 6.8 $\Omega$ cm^2^ and stabilized at a value of about 5.3 $\Omega$ cm^2^ after 165 h of aging. From then on, the membrane resistance declined slightly to a final value of 4.8 $\Omega$ cm^2^. The cathode charge transfer resistance increased in the low humidity regime and recovered to nearly the original values measured before this treatment (Fig. 6b). Interestingly, the resistance exhibited from then on rather stable values of about 25 $\Omega$ cm^2^. After approximately 400 h of aging, the value started to slightly decrease again to the final value of 21 $\Omega$ cm^2^. A similar behavior was observed for the Warburg impedance during the first aging hours (Fig. 6d). An inital decline of the impedance at the beginning was followed by a sharp increase under low humidity conditions and relaxation to the original values under normal humidities. However, after approximately 200 h, a continuous increase from about 1.0 $\Omega$ cm^2^ to a final value of about 1.5 $\Omega$ cm^2^ was recorded. The cathode capacitance was reduced by one order of magnitude in the low humidity regime, with a drop from 24 to 2.5 mF (Fig. 6e). The capacitance recovered fully to the starting value after turning on the humidifier. Interestingly, no further trend could be observed afterwards, i.e. the capacitance remained until the end rather stable with values oscillating around 26 mF. The exponent of the CPE capacitance showed a slightly increasing trend, rising from average values of 0.79 to 0.80 (Fig. S1).

### Reaction gas analysis

**Figure 7:**
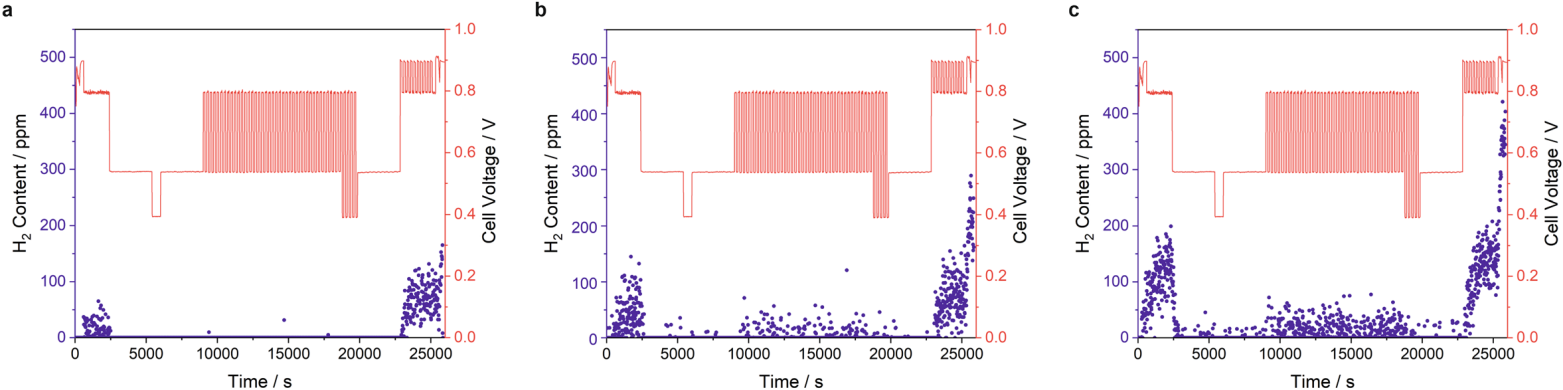
H_2_ content of the cathode exhaust gas and corresponding measured cell voltages recorded during AST cycle number (**a**) 4 (21–28 h of aging), (**b**) 100 (710–717 h) and (**c**) 124 (882–889 h). Shown are the spectra recorded during the 95 ^∘^C/80 %*RH* section of the AST program.

During the aging cycles, the cathode outlet gas was analyzed using mass spectrometry (MS). Representative results are depicted in Fig. 7 for different aging stages. Cycles of the AST section with 95 ^∘^C and 80 %*RH* are shown, as the hydrogen content in the outlet gas was highest under these conditions. It can be observed that the hydrogen content in the cathode output was highest at OCV conditions during all tests. At the beginning of aging, during the dynamic cell voltage changes as well as in the steady-state sections with lower cell voltages, nearly no hydrogen penetration was detectable. This changed with increasing aging time. While approximately 50 ppm H_2_ was measured in the dynamic sections of cycle 100, the values oscillated around 75 ppm in cycle 124. The maximum H_2_ leakage recorded at OCV increased over time from about 50-150 ppm in cycle 4 (21–28 h of aging), over 150-300 ppm in cycle 100 (710–717 h) up to 200-400 ppm in cycle 124 (882–889 h).

### Reaction water analysis

**Figure 8:**
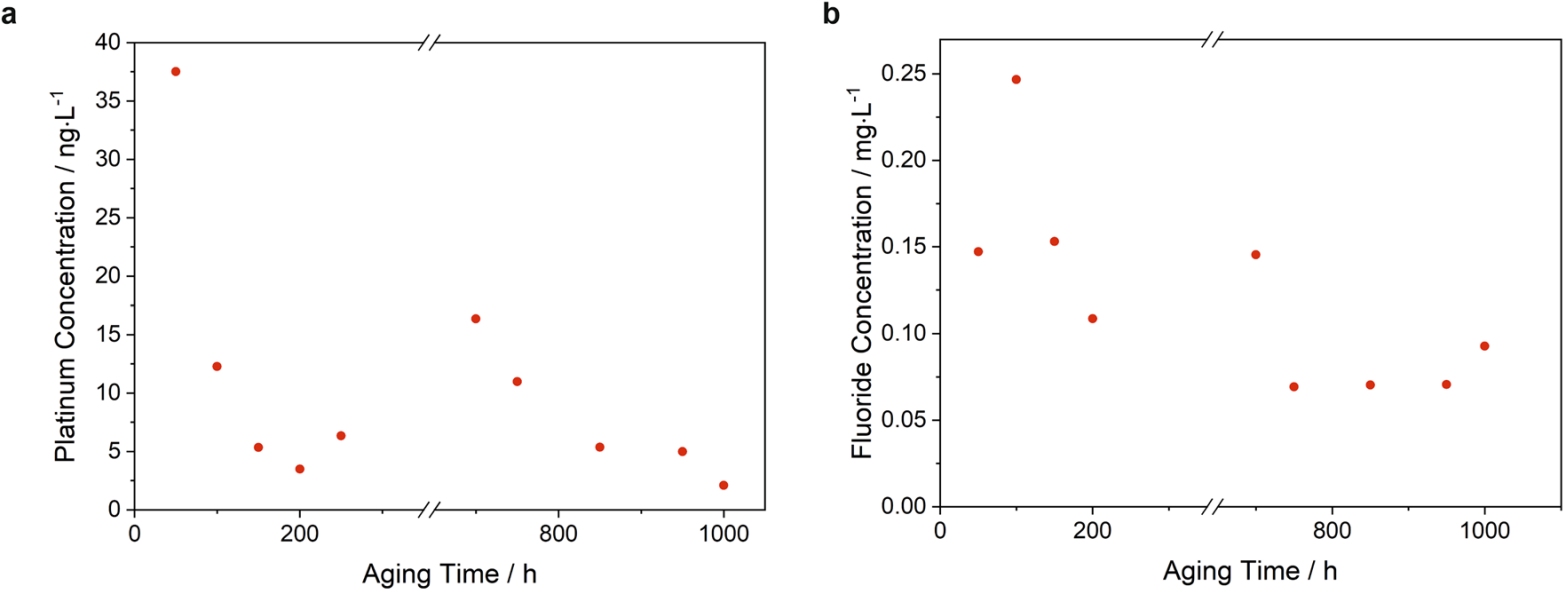
Platinum (**a**) and fluoride (**b**) concentration of the condensed water of the cathode fuel cell exhaust measured during the AST program after indicated aging times.

After defined time intervals of the AST program, the condensed water from the fuel cell exhaust was removed from the respective collection vessel and analyzed for platinum using polarography. The individual measurement results were plotted against aging time (Fig. 8a). At the beginning of the AST program, a platinum concentration of 37.5 ng/L was found in the condensed cathode outlet water. Thereafter, the concentration decreased to 3.5 ng/L after 200 h. After 600 h aging time, the value increased to 16.4 ng/L and decreased finally to 2.1 ng/L after 1000 h.

In addition, the fluoride content of the condensed fuel cell cathode exhaust water was analyzed by ion chromatography (IC). The obtained fluoride concentrations are shown in Fig. 8b. As a result, fluoride concentrations between 0.07 and 0.25 mg/L were obtained. In the course of the accelerated aging test, the fluoride emission initially remained at a relatively high level between 0.1 and 0.15 mg/L for most samples and tended to decrease after about 700 h of aging to values below 0.1 mg/L.

### Microscopy

#### X-ray fluorescence microscopy ($\mu$-XRF)

**Figure 9:**
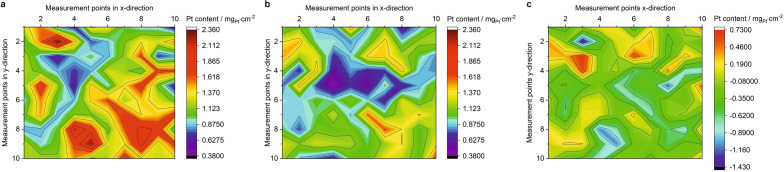
$\mu$-XRF platinum mapping of the investigated fuel cell MEA before (**a**) and after 1000 h of AST aging (**b**). In (**c**) the aging induced local change of the platinum content is visualized. Note that all images show the entire $5\times 5\,\hbox {cm}^2$ area of the MEA.

Before the beginning and after the end of the AST program, $\mu$-XRF was used to map the platinum content of the entire $5\times 5\,\hbox {cm}^2$ area of the investigated MEA. This was done to determine the absolute platinum loading as well as the platinum distribution on the MEA as a function of aging. As can be seen in Fig. 9a, the platinum distribution of the MEA was relatively inhomogeneous at the beginning. In general, local platinum concentrations were found in a range between 0.57 and 2.36 mg_Pt_/cm^2^. The average loading over all 100 measurement points was 1.24 mg_Pt_/cm^2^.

After aging for 1000 h (post-mortem), a significant platinum loss became apparent (Fig. 9b). Now, the platinum content of the MEA ranged from 0.38 to 1.61 mg_Pt_/cm^2^. An average platinum loading of 1.04 mg_Pt_/cm^2^ was calculated, corresponding to a platinum loss of 0.20 mg_Pt_/cm^2^ (corresponding to a relative loss of 16 %). In order to draw more conclusions about the change in platinum distribution, the absolute change in platinum content at each measurement point is illustrated in Fig. 9c. When looking at the location-dependent change in the platinum content, it becomes obvious that in many parts of the MEA the platinum loading has decreased. Nevertheless, an increase in platinum could also be observed in some areas. It can be seen that platinum has been removed mainly at positions where previously high concentrations of platinum were present. At positions with originally low platinum content, an increase was observed after aging. The difference between the lowest and highest platinum content before aging was 1.79 mg_Pt_/cm^2^, while this value decreased to 1.23 mg_Pt_/cm^2^ after aging, indicating a homogenization of the platinum loading on the MEA.

#### Scanning and transmission electron microscopy (SEM and TEM)

**Figure 10:**
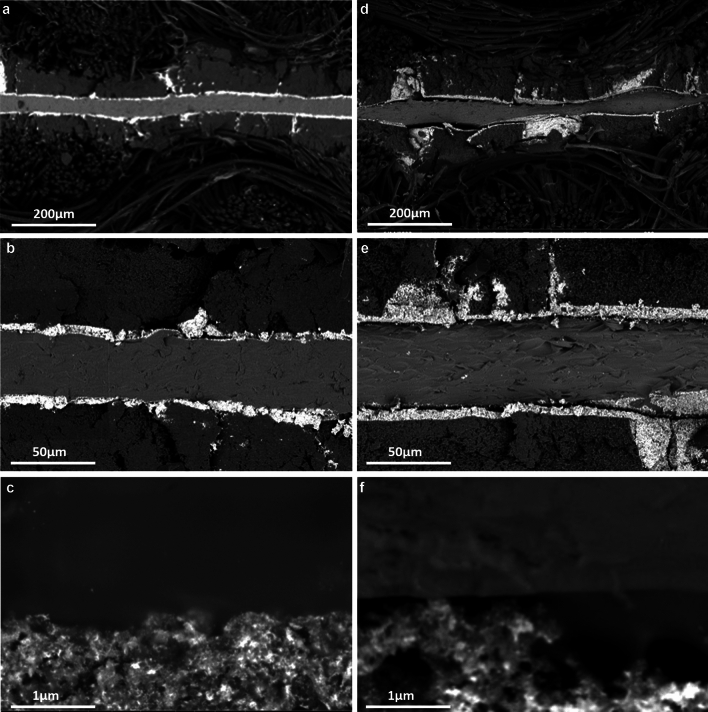
Representative cross-sectional SEM images in different magnifications of a pristine MEA (**a**–**c**) and of the MEA aged for 1000 h in the AST program (**d**–**f**). The cross-sections were prepared with the cathode side at the top and the anode side at the bottom of the images.

In order to compare the microstructure and morphology of the MEA before and after aging, cross-sections of a pristine and of the 1000 h AST aged MEA were investigated by SEM (Fig. 10). As for the pristine MEA, a typical thickness of 35-40 $\upmu$m can be deduced for the membrane. The porosity of the membrane appears to be rather smooth and no larger pores or other pecularities such as cracks or pinholes can be identified in the images. The supported catalyst is characterized by a thin layer with a thickness of about 5 $\upmu$m closely attached to the membrane on both anode and cathode side. However, bright spots that can be assigned to platinum particles can also be found at a greater distance from the membrane. The membrane itself appears to be free of platinum particles. At some minor positions a slight delamination of the CL from the membrane can be recognized. However, this might be a preparation artifact due to the rather high mechanical stress associated with the cutting of the MEA.

After aging, the microstructure is strongly modified. The membrane is now characterized by a strong periodic modulation of its thickness, as can be seen in Fig. 10d. Parts of the membrane are with approximately 20 $\upmu$m significantly thinner than in the pristine MEA, whereas other parts are with up to 55 $\upmu$m thicker than before. Moreover, the CL is now over large regions delaminated from the membrane. This phenomenon can be observed on both anode and cathode side. Comparing Fig. 10b and e indicates that also the porosity of the MPL has changed due to generally much larger pores. Figure 10f also gives the impression that the membrane is much rougher and more irregularly patterned, which was not observed in the pristine MEA to such an extent (Fig. 10c). One interpretation could be that the elastic properties of the membrane have changed and that the breaking in liquid nitrogen for the preparation of the cross-cut samples has led to somewhat rougher surfaces in the spent MEA. In addition, platinum particles are now also found inside the membrane of the aged MEA (Fig. 10e). However, it cannot be ruled out that this is a preparation artifact during cross-cutting, which is due to very loosely bound and therefore easily mechanically displaced platinum particles. Overall, no obvious differences were found between the anode and cathode sides with regard to the effects of aging.

TEM images of the spent MEA reveal a stronger aggregation of platinum nanoparticles than of the pristine MEA (Fig. S2). The pristine MEA is generally characterized by smaller platinum nanoparticles and—although not completely homogeneously dispersed—the carbon is generally very highly loaded. The comparison of STEM images recorded with a surface sensitive secondary electron detector and High-Angle Annular Dark-Field (HAADF) images indicates that many well separated smaller platinum nanoparticles on top and bottom of the carbon particles give rise to the strong z-contrast (Fig. S3). The carbon support itself appears to be rather unaffected from aging.

## Discussion

For a coherent discussion of the results, the fuel cell behavior is divided into the following characteristic stages during the aging program: break-in phase (phase at the beginning of the AST program), phase with humidifier turned-off, phases between 100 and 400 h, 400 and 600 h, and after 600 h of aging (end-of-life phase).

### Break-in phase

The first hours during the AST program are characterized by an increasing cell voltage over the entire range of the polarization curve, i.e. in the OCV, activation, ohmic and mass transport region. This phenomenon is typical for the so-called break-in phase (also called activation, incubation or conditioning phase). Usually, this is assigned to the hydration of the membrane, the removal of impurities from the manufacturing process and the creation of transfer passages for the reactants to the catalyst^40^. This interpretation is supported by the initial increase of the ECSA and the decreasing Warburg impedance indicating a higher catalytic activity and improved gas transport through the MEA, respectively. Simultaneously, the membrane and ohmic resistance increased, as measured by LSV, CI and EIS. This can be also exlained by the increased hydration of the membrane which gives rise to a stronger electrically insulating behavior. The hydration is usually associated with a swelling of the membrane, a better contact to the CL and a higher proton conductivity. The decreased charge transfer resistance of the cathode as measured by EIS supports the idea of an improved catalyst-membrane interface. In general, the polarization curve is very well comparable with measurements on similar MEAs with NR-212 membrane and comparable platinum loadings under similar experimental conditions^27,28^. The maximum ECSA value (56 m^2^/g_Pt_) is also very similar to the 50 m^2^/g_Pt_ found for a comparable Nafion™ 212 based MEA^32^. The high platinum concentration in the product water at the beginning of the AST program can be explained by a removal of platinum not or only very loosely bound to the support. SEM images show indeed that a rather high platinum concentration can be found outside of the CL. This interpretation is also supported by the XRF analysis which gives evidence for a very inhomogeneous distribution of platinum over the entire MEA at the beginning of life.

### Phase with turned-off humidifier

The low humidity induces a very large power and cell voltage drop. This affects in particular the ohmic and mass transport region, while the OCV is only slightly affected. It can be expected that the dehydration of the membrane under dry conditions causes a decreasing proton conductivity and a contraction of the membrane. The reduced ion conductivity is reflected by the increase of both ohmic resistance as measured by CI and membrane resistance as deduced from EIS. Interestingly, the short-circuit resistance calculated from LSV decreases under low humidities, which can be explained by a loss of insulating water molecules. The falling ECSA and capacitances and rising charge transfer resistances point to a weakened interface between catalyst, membrane and MPL. The increased Warburg impedance can be explained by an impeded gas transport across this weakened GDL/MPL/catalyst interface. It is assumed that the severe mass transfer losses result from the swelling and shrinking of the PEM under strongly changing humidities, which places mechanical stress on the entire MEA finally leading to a weakened interface. In addition, the decreased hydrogen crossover current evidences a lower hydrogen permeation through the membrane. This might be due to the generally impeded gas transport through the MEA as explained before. Interestingly, many of the measured parameters show a full recovery after again turning on the gas humidifier, indicating that the involved processes are reversible. Basically only the cell voltage in the mass transport range does not recover, i.e. the high power and high current performance associated with a high gas transport is permanently deteriorated. This is not reflected in the Warburg impedance, which shows a nearly complete recovery. However, since the EIS was measured at low current densities, i.e. not under mass transport limitation, the effect did probably not show up under the applied measurement conditions. The irreversibly deteriorated mass transport under high loads might be due to a diminished hydrophobicity of the MPL and/or to a deteriorated interface between GDL and CL caused by the intermediate dry and hot conditions. A higher porosity of the MPL after aging could be evidenced by the SEM investigations at the end of life.

### Phase between 100 and 400 h of aging

This phase is characterized by a rather stable performance of the PEMFC. The cell voltage does not change significantly during this period. Only the OCV starts to drop slightly after approximately 200 h of aging. Parameters associated with the catalytic activity such as the charge transfer resistance and capacitance remain constant. No clear trends can be observed for the H_2_ crossover current, the ohmic resistance as measured by CI and the membrane resistance as measured by EIS. Obviously, gas permeation and proton conductivity of the membrane remain stable in this aging phase. However, both the active surface (ECSA) and the short-circuit resistance as measured by LSV are continuously decreasing. This together with the still measurable platinum content and the very high fluoride concentration of the collected exhaust water indicate a continuous degradation of the catalyst and the membrane (or ionophors) in the MEA also in this stage of the AST program.

### Phase between 400 and 600 h of aging

This time period appears to be the turning point between the previously observed rather stable performance and an accelerated degradation. After approximately 400 h, the OCV exhibits a significant drop, while the ohmic and mass transport regions still seem rather unaffected. The parameters associated with the membrane’s state-of-health clearly indicate a degradation. The increasing H_2_ crossover current proves a severe gas permeability, which can be also detected in the exhaust gas by MS. Hence the falling OCV can be directly assigned to a higher partial pressure of hydrogen at the cathode which gives rise to a lower cathode potential. This OCV drop below 0.9 V due to an increased hydrogen permeability is typical for the NR-212 membrane and was also reported by other groups, e.g. after 400 h of idling conditions^28^ or after artificial aging by the ion-exchange method^27^. Strong irreversible OCV decay rates between 0.083 and 0.141 mV/h upon OCV durability testing at 90 ^∘^C cell temperature were also reported for other catalyst-coated membranes and as well assigned to an increased hydrogen crossover^41^. The obtained OCV degradation rate of 0.1 mV/h upon AST aging of the MEA tested in our study is hence well in this range. The decreasing ohmic resistance measured by CI points to a slightly increased proton conductivity, while the decreasing short-circuit resistance deduced by LSV indicates a higher electronic conduction of the membrane. This can be also interpreted as indicators for an increasing membrane porosity. The ECSA starts to stabilize in this period of time. A peak of the platinum concentration in the product water after 700 h gives rise to the assumption that there is a final release of a larger fraction of platinum at this point. Afterwards, the platinum content decreases which might explain the stabilization of the ECSA value since all loosely bound platinum in the MEA has been released.

### Phase between 600 h of aging and end of life

After 600 h of aging the OCV drops even more significantly and the differences in cell voltage between the different temperature and humidity conditions become very large. The increasing voltage oscillations can be also observed for the ohmic and mass transport region. The strongly increasing membrane permeability indicated by the increasing H_2_ crossover current and the high H_2_ concentration in the cathode exhaust gas as well as the further falling electronic resistance as measured by LSV indicate that the membrane degrades at an ever increasing rate, which finally defines the end of life of the PEMFC. After 1000 h, various parameters of the polarization curve decreased by more than 10 % (peak power densitiy by 12.1 %, OCV by 10.0 %, cell voltage in mass transport region by 19.2 %), which is defined as end of life of the fuel cell by the DoE. Moreover, the crossover current density increased to more than 10 mA/cm^2^, a value which is also associated with the end of life of the membrane by the manufacturer. After approximately 900 h, a further decrease of the active surface is observed. At the end, the active surface of the catayst decreased by more than 50 % during the entire AST program and about 16 % of the platinum got lost. The DoE defines the end of life of the CL after more than 40 % loss of the original ECSA^42^, which is here the case. Typically, the capacitance of aging electrodes as deduced from EIS measurements decreases and the CPE exponent moves from 0.8 towards 1. At a value of 0.8, a porous surface structure of the electrodes can be assumed^43^. Over time, catalyst particles detach, the porosity of the catalyst decreases and the exponent approaches the value 1. The value 1 corresponds to a capacitor with a smooth structure. This effect is in principal also observed for the here investigated MEA, even though the total capacitance remains rather constant after its initial increase at the beginning of the AST program. The strongly enhanced delamination of the CL from the membrane—as another obvious sign of catalytic deactivation—is clearly evidenced in the SEM images. The acceleration of the aging at the end of the program likely comes from the formation of hydroxide radicals at hydrogen/oxygen fronts with the increasing H_2_ permeation through the membrane. The radicals can attack the C–F bonds of the membrane as schematically shown by the following equations^44^:

1 $$\begin{aligned}{} & {} {\mathrm{R-CF}_{2}\textrm{COOH}} + {\textrm{OH}^\cdot } \longrightarrow {\mathrm{R-CF}_{2}^{\cdot }} + {\textrm{CO}_{2}} + {\textrm{H}_{2}\textrm{O}} \end{aligned}$$

2 $$\begin{aligned}{} & {} {\mathrm{R-CF}_{2}^\cdot} + {\textrm{OH}^\cdot } \longrightarrow {\mathrm{R-CF}_{2}\textrm{OH}} \longrightarrow {\textrm{R}-\textrm{COF}} + {\textrm{HF}} \end{aligned}$$

3 $$\begin{aligned}{} & {} {\mathrm{R-COF}} + {\textrm{H}_{2}\textrm{O}} \longrightarrow {\mathrm{R-COOH}} + {\textrm{HF}} \end{aligned}$$

The generation of HF as further evidence for this kind of membrane degradation could be proven by the high flouride content of the exhaust water. The processes reinforce this way each other and cause an exponential increase of the permeability. The degradation process can also be catalyzed by platinum that might have migrated into the membrane. From our water analysis data (and an estimated total volume of 15 L of collected (product and humidifier) water during the entire AST program), an average fluoride release rate (FRR) at the cathode of 0.0039 $\upmu$mol/cm^2^/h can be calculated. This rate is approximately two orders of magnitude lower than the reported FRR of Nafion™ 212 membranes directly aged with Fenton‘s reagent^29,30^. This is comprehensible since this scenario is a much more extreme chemical deactivation treatment. The FRR of the MEA investigated in our study is however well in the range of the FRR obtained by testing an entire MEA with a Nafion™ 112 membrane under different current densities^31^.

Interestingly, the hydrogen content of the cathode exhaust gas in this late deactivation phase is highest under OCV, idling and start/stop conditions, i.e. at low current densities. This can be explained by the high partial pressure of hydrogen at the anode/membrane interface under low loads (when nearly no hydrogen is consumed) which causes the highest hydrogen gradient between anode and cathode and hence a high driving force for hydrogen to penetrate the membrane. The lowest voltages are observed in the OCV, activation, ohmic and mass transport region under high temperature and high humidity conditions, while the highest cell voltage is maintained under high temperature and lower humidity. Obviously, the phase of highest hydrogen crossover does also determine the point of the weakest performance of the fuel cell. The swelling of the membrane under high humidities (and high temperatures) is likely to further increase the pore size and the channel diameter inside the membrane leading to the enhanced hydrogen crossover.

MS on-line measurements of the CO_2_ content of the cathode exhaust gas exhibited only a low constant base line independent of the aging program. Hence a significant carbon corrosion due to total oxidation of the carbon support could not be evidenced.

SEM images indicate a less smoother membrane at the end of life. However, no obvious cracks are observed. Obviously, the increased gas permeability is mainly caused by chemical attacks inside the membrane destroying the perfluorinated hydrocarbon network on a molecular scale as discussed before. This is also supported by the continuous release of fluoride ions as detected in the reaction water. The modulation of the membrane thickness also observed in the SEM images is probably due to the compression of the MEA between the flow channels of the end plates during installation in the fuel cell test fixture. However, this fixing did obviously not induce significant mechanical cracks or pinholes. Moreover, TEM images reveal a pronounced aggregation and growth of platinum particles after end of life which is in agreement with the significantly reduced ECSA and platinum migration as observed by XRF. It is well known that platinum can be electrochemically oxidized at high local electrical potentials in fuel cells^8^:

4 $$\begin{aligned} {\textrm{Pt}} \longrightarrow {\textrm{Pt}^{2+}} + {2\textrm{e}^{-}}. \end{aligned}$$

Alternatively, a chemical oxidation with water and the formation of a surface hydroxide and oxide is possible:

5 $$\begin{aligned}{} & {} \mathrm{{Pt}} + {\textrm{H}_{2}\textrm{O}} \longrightarrow {\text {Pt(OH)}} + {\textrm{H}^{+}} + {\textrm{e}^{-}} \end{aligned}$$

6 $$\begin{aligned}{} & {} {\text {Pt(OH)}} \longrightarrow \mathrm{{PtO}} + {\textrm{H}^{+}} + {\textrm{e}^{-}}. \end{aligned}$$

In addition, cathodic dissolution of platinum has been also associated with the electrochemical reduction of PtO_2_^8^:

7 $$\begin{aligned} \mathrm{{PtO}}_2 + {\textrm{4H}^{+}} + {\textrm{2e}^{-}} \longrightarrow {\textrm{Pt}^{2+}} + {\textrm{2H}_{2}\textrm{O}}. \end{aligned}$$

The platinum oxide can diffuse via the surface from the CL to the interface with the PEM of the fuel cell^45^. There, molecular hydrogen from the anode crossing the membrane can reduce Pt^2+^ again to elemental platinum, which can redissipate or agglomerate subsequently to (larger) nanoparticles^11^—as evidenced in the TEM images. Interestingly, a significant in-plane movement of platinum over large distances during aging as observed in our $\mu$-XRF images of the spent MEA was also reported by Khedekar et al.^46^ This indicates that Ostwald ripening and particle coalescence are not restricted to local effects. Alternatively, platinum ions are released with the humid exhaust gas stream^10,47^—as also observed by the platinum content in the analyzed exhaust water. Based on this data we calculated a total loss of about 0.2 $\upmu$g of platinum in the collected cathode exhaust water. This value corresponds to an average platinum dissolution rate (PtDR) of $2\times 10^{-15}$ g/cm^2^/h. This rate is approximatetely one order of magnitude lower than the PtDR reported for different platinum forms (sheet, wire, film, C-supported Pt nanoparticles) after polarization at potentials close to or above 0.9 V_SHE_ in perchloric acid solution^8,48^, but higher than the values reported for a 100 W PEM fuel cell stack aged in dynamic and high load stress tests^47^. According to our XRF investigations, a total platinum loss of 5 mg was measured. This comprehends the loss on both anode and cathode side. The comparison of these figures shows that less than 0.01 % of the lost platinum is released with the exhaust water in a soluble form if the loss is evenly distributed on both electrode sides.

Moreover, TEM reveals that the carbon support appears to be hardly affected by aging. This would be in agreement with the MS data and the absence of CO_2_ as indicator for carbon corrosion. Since no potentials higher than the OCV were applied, (irreversible) carbon oxidation is obviously not induced by this AST protocol.

### Application of the AST to a high-performance MEA for HDV applications

As already stated at the beginning of this publication, we tested the AST also on an advanced commercial fuel cell MEA specifically manufactured for demanding HDV applications under the same conditions. As a result, after 1000 h AST aging, a decrease in peak power density (from > 800 mW/cm^2^) by about 10 % and of the ECSA by about 25 % was measured, while nearly no change of the hydrogen crossover current density (2 mA/cm^2^) was observed. The OCV decreased only slightly from 0.95 to 0.94 V. This proves the superior durability of this sample. After 2070 h of AST aging, the peak power density and the ECSA decreased by nearly 20 % and 40 %, respectively, hence defining the end of life of this MEA. The membrane remained rather stable as proven by the only sligtly increased hydrogen crossover current density (3 mA/cm^2^) and the slightly reduced OCV (0.92 V). This shows that this AST program is also suitable to test and distinguish high-performance PEMFC components. The acceleration aging factor of this test will be clarified in the future by comparing the results with the aging of corresponding fuel cells in standardized (e.g. by DoE) AST and truck field tests.

### Conclusions

In summary, the newly developed dynamic AST and analysis program has proven to be a very useful tool to analyze the failure modes during the operation of a single PEMFC under conditions comparable to characteristic HDV driving scenarios. The separation of the AST into different stress sequences in terms of voltage, temperature and humidity and the introduction of analysis cycles, which also serve as recovery steps, allow the identification of causal relationships between stressors and affected components of the MEA. As for the investigated PEMFC, the end of life was reached after 1000 h of aging with regard to the indicators OCV, maximum power, hydrogen crossover current and ECSA reduction. The negative effects of an intermediate low-humidity phase proved to be reversible, with the exception of the performance in the mass transport limited high current density range. The results also indicate that the performance of the investigated MEA could have been improved by a combination of high temperature and moderate humidity treatment at the end of life. This is due to the lower hydrogen permeation under these conditions, which should also result in a lower degeneration rate. Since hydrogen penetration is greatest at low current densities, i.e. under OCV and idling/low load conditions, this should also be avoided in order to extend the service life. As a consequence, these findings show that this AST and analysis program can help to identify operating parameters in order to extend the service life of the fuel cell. Moreover, this information can be also used to accelerate the aging of the MEA if a shorter AST is desired.

## Methods

### Instrumentation for accelerated stress test and electrochemical characterization

All electrochemical measurements and the accelerated stress test were run on the commercial 850e Fuel Cell Test System (Scribner Associates, Inc.) including the 881 Frequency Resistance Analyzer, the two potentiostats 850e and 885 and a PEM fuel cell fixture. The tested MEA for this study was a 5-layer Hydrogen Air MEA (Fuel Cell Store, Texas, USA) with an active area of 25 cm^2^ and a nominal platinum catalyst loading of 0.5 mg_Pt_/cm^2^ (60 wt% Pt on Vulcan (carbon)) on each side. The cathode and anode layers were separated by a Nafion™ PFSA NR-212 membrane (nominal thickness 50.8 micrometers). As gas diffusion layers carbon cloth with microporous layer W1S1010 (woven carbon fiber cloth; nominal thickness 365 micrometers) was used on both sides. The test system was operated with hydrogen (5.5, Rießner Gase, Lichtenfels, Germany), filtered air (CO_2_ free, CO free, H_2_O free and particulate free) and nitrogen (5.0, Rießner Gase, Lichtenfels, Germany). During the accelerated aging test, flow rates of 0.013 L/min/A for hydrogen and 0.052 L/min/A for air were applied. This corresponds to a stoichiometry of $\lambda _\text {anode}$ = 1.5 and $\lambda _\text {cathode}$ = 2.5. The minimal flow rates were set to 0.026 L/min at the anode and 0.104 L/min at the cathode. The pressure for the entire measurement protocols was set to 0 bar_g_ on both electrode sides using the back-pressure unit of the test system.

### Protocol for electrochemical characterizations

Electrochemical cell diagnostics was performed in regular intervals after every single applied voltage cycle. All electrochemical analysis cycles were recorded at 70 ^∘^C cell temperature and 80 % relative gas humidity. The gases applied differed depending on the measurement.

#### Polarization measurements

In order to record the polarization curve, the anode was operated with hydrogen and the cathode with air. The anode/cathode stoichiometry was set at 1.5/2.5. Assuming an oxygen content of 21% in air, the flow rates were 0.013 L/min/A for hydrogen and 0.052 L/min/A for air. The measurement was performed in two steps. In the first one, the current was ramped up from 0 (OCV) to 1.9 A with a scan rate of 50 mA/s. In the second step, a current of 1.9 A to a maximum of 50 A was then applied, with a scan rate of 200 mA/s. Measurements were stopped automatically when the voltage fell below 0.3 V.

#### Linear sweep voltammetry

For the LSV measurement, the anode side was purged with hydrogen and the cathode side with nitrogen. The reactant flow rates were 0.2 L/min for hydrogen and 0.1 L/min for nitrogen. An initial voltage of 0.05 V was applied, which was increased linearily to 0.5 V at a scan rate of 1 mV/s. During the measurement, the generated current density was measured. Since only nitrogen is present at the cathode, the generated current is due to the oxidation of permeated hydrogen. The current initially increases rapidly and reaches a plateau at about 0.15 V (cf. Fig. S4). At this point, all of the permeated hydrogen is oxidized and current generation is limited by mass transport. If the current then continues to rise linearly with the applied voltage, an electrical short circuit is present. This means that electrons are able to diffuse through the membrane despite its insulating properties, thus creating a short circuit. According to Ohm’s law, the slope is equal to the reciprocal value of the short-circuit resistance.

#### Cyclovoltammetry

During the measurement of the whole cyclic voltammogramm, a constant flow of 0.2 L/min hydrogen was used on the anode side, nitrogen was applied on the cathode side. The entire voltammogramm was recorded in the potential range of 0.05-1 V with a scan rate of 30 mV/s. In order to determine the electrochemically active surface, the area under the curve was calculated under the peaks associated with hydrogen desorption as shown in Fig. S5.

#### Current interruption

Hydrogen at the anode with a flow rate of 0.013 L/min/A and air at the cathode with a flow rate of 0.104 L/min/A were applied. In the first step, a current of 2 A was kept constant for 120 s. The current was then reduced to 0 A for 10 s. The difference was formed from the value of the voltage directly before and after the current reduction. The ohmic resistance was calculated from the quotient of this voltage difference and the current applied before reduction to 0 A.

#### Electrochemical impedance spectroscopy

The gases hydrogen at the anode (${\dot{V}}$=0.013 L/min/A) and air at the cathode (${\dot{V}}$=0.052/L/min/A) were applied during recording EIS. The spectra were recorded in the potentiostatic mode in the frequency range of 0.1-10,000 Hz at an amplitude of 10 mV. The frequency was increased logarithmically with ten steps per decade. For the equivalent circuit fit, the equivalent circuit model shown in Fig. S6 was used.

### Reaction gas analysis

The mass spectrometer QGA (Hiden Analytical, Warrington, UK) was connected to the cathode outlet down-stream of the water condensation tank by stainless steel tubings and fittings to measure the reaction gases. A mass spectrum in the range of 1-60 amu was recorded with MASsoft10 software every 30 s. After evaluation, all detected gases were embedded into the Hiden QGA software to record the time evolution of the gas fractions.

### Product water analysis

The cathode exhaust gas was fed through stainless steel pipes into a stainless steel tank to condense out the water. The collected water was periodically sampled and analyzed for platinum and fluoride.

#### Polarographic platinum analysis

The analysis procedure to quantify platinum in the fuel cell reaction water by polarography is described in detail by Birkner et al.^47^ The analysis was performed in a standard three-electrode cell on a 797 VA Computrace voltammeter (Metrohm, Herisau, Switzerland) equipped with a multimode electrode, a glassy carbon electrode as auxiliary electrode and an Ag/AgCl electrode (3 M KCl) as reference electrode. The platinum concentration was determined using standard addition.

#### Ion chromatography

The samples were analyzed for fluoride ions using the ion chromatography system 883 Basic IC plus (Metrohm, Herisau, Switzerland). The setup was equipped with a Metrosep A Supp 4-250/4.0 anion exchange column. 1.8 mmol/L sodium carbonate (p.a., Carl Roth, Karlsruhe, Germany) and 1.7 mmol/L sodium hydrogen carbonate (p.a., Carl Roth, Karlsruhe, Germany) served as eluent. The flow rate was set to 1.0 mL/min. The run time was about 14 min and the injection volume was 1.5 mL. Data acquisition was performed by using the MagIC Net software (Metrohm, Herisau, Switzerland). A six-point calibration curve of 0.05-0.5 mg/L was recorded before the measurements. Stock solutions were prepared from a 1,000 mg/L fluoride standard (p.a., Carl Roth, Karlsruhe, Germany) and milli-Q water.

### Microscopy

#### X-ray fluorescence microscopy

XRF measurements were run on the XGT-9000 $\mu$-X-ray fluorescence spectrometer (HORIBA, Oberursel, Germany). A calibration was carried out for quantification of platinum as illustrated in Fig. S7. For this purpose, electrode standards (platinum on carbon cloth) with three different platinum loadings (Fuel Cell Store, Texas, USA) were used (0.03 mg/cm^2^ 20 % Platinum on Vulcan-Carbon Cloth Electrode, 0.3 mg/cm^2^ 40 % Platinum on Vulcan-Carbon Cloth Electrode, 0.5 mg/cm^2^ 60 % Platinum on Vulcan-Carbon Cloth Electrode). To simulate MEAs with different platinum loadings for the calibration, a Nafion™ NR212 membrane (DuPont, USA) was placed between two electrode standards. This way, all possible configurations of two electrode standards with equal or different platinum loadings were assembled. XRF spectra of the this way assembled standards and of the to be investigated MEA were measured under vacuum. The electrode standard with lowest platinum loading was—if different from the second standard—always put on top of this assembly. The intensities of the Pt L_α_ radiation were used for the calibration. Spectra at 100 measurement points ($10\times 10$ point array) were recorded over the entire $5\times 5\,\hbox {cm}^2$ area of the investigated samples. Each measurement point was recorded in transmission mode using a 15 *μ*m high-intensity polycapillary with a duration of 120 s and a voltage of 50 kV. In addition, a mapping recording was made. Here, 256 pixels in x-direction and 200 pixels in y-direction were measured over the entire surface. The measurement duration for one pixel was 50 ms. The potential and the capillary were identical to the spectrum recording, the current was adjusted automatically. No filter was used for the measurements.

#### Electron microscopy

SEM images were recorded using a Thermo Fischer Helios Nanolab 600i DualBeam FIB-SEM operated at acceleration voltage of 4 kV or 5 kV. Cross-sections for SEM imaging were prepared by cutting the MEA at liquid nitrogen temperature.

For TEM imaging, pieces of the MEA were embedded in epoxy resign and cut by microtome into thin slices for TEM imaging. TEM images were recorded using a JEOL F200 transmission electron microscope operated at 200 kV.

### Supplementary Information

Supplementary Figures.

## Data Availability

Data sets used and/or analyzed in this study are available from the corresponding author upon reasonable request.
