# Supplementary material for: Dynamic accelerated stress test and coupled on-line analysis program to elucidate aging processes in proton exchange membrane fuel cells
Source: Sci Rep. 2024 Feb 18;14:3999. doi: 10.1038/s41598-024-54258-8 (PMC10874950; doi:10.1038/s41598-024-54258-8)
Supplement: Supplementary file 1 — Supplementary Figures. [file 41598_2024_54258_MOESM1_ESM.pdf]

Supplementary Information:

Dynamic accelerated stress test and coupled on-line analysis program to elucidate aging processes in proton exchange membrane fuel cells

Lena Birkner[1,2], Michael Foreta[2], Ali Rinaldi[3], Anton Orekhov[3], Marc-Georg Willinger[3], and Maik Eichelbaum[1,*]

[1]Nuremberg Institute of Technology, Institute for Applied Hydrogen Research, Electro- and Thermochemical Energy Systems (H2OHM), Nuremberg, 90489, Germany

[2]MAN Truck & Bus SE, Material Technology and Applied Chemistry (EOMC), Nuremberg, 90441, Germany

[3]Technical University of Munich, Chair of Electron Microscopy, Garching, 85748, Germany

[*]maik.eichelbaum@th-nuernberg.de

Supplementary Information

Supplementary Tables and Figures

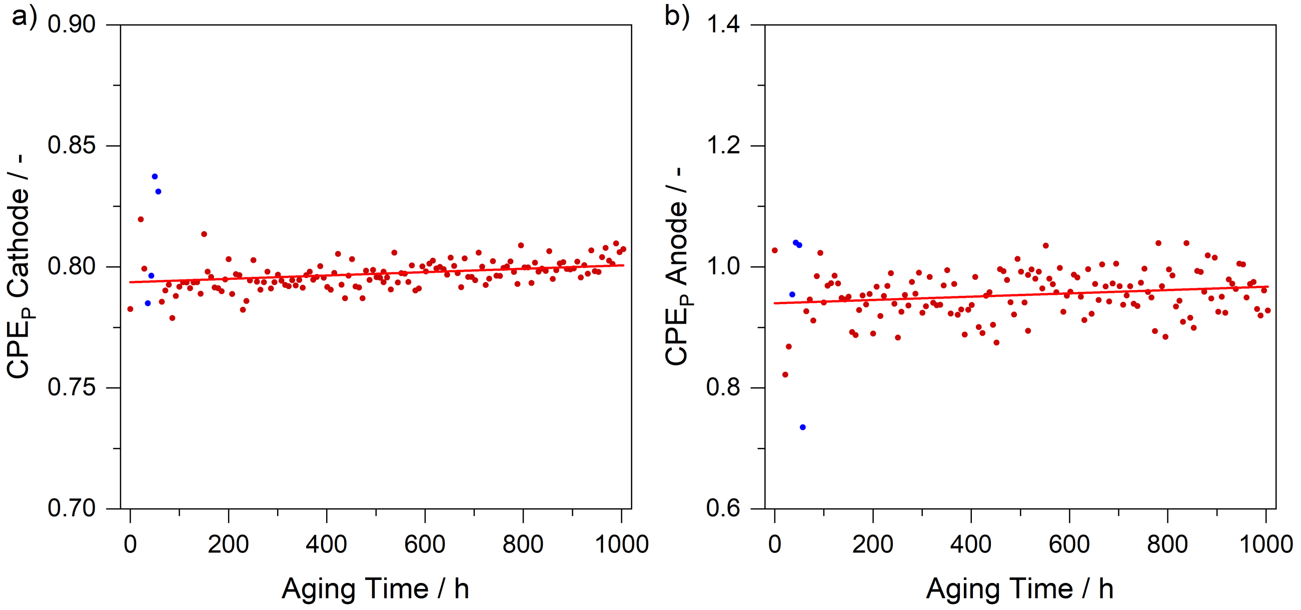

Figure S1. Exponents of the CPE capacitances of the cathode (a) and anode (b) as deduced from equivalent circuit fits of EIS data. The lines are the results of linear regressions of the data points. Blue data points represent measurements with turned-off humidifier.

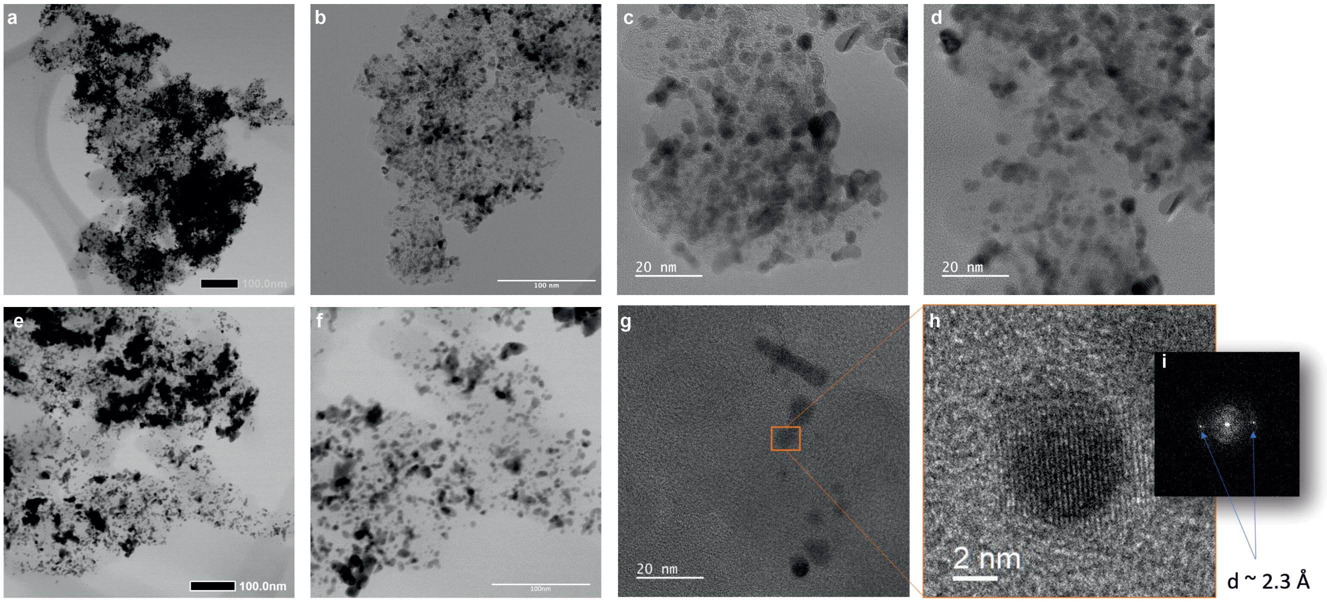

Figure S2. Representative Transmission Electron Microscopy (TEM) images of the platinum catalyst and carbon support from a pristine (a-d) and 1000 h AST aged (e-h) MEA. i) Selected Area Electron Diffraction (SAED) pattern of the nanoparticle depicted in h. The identified interplanar spacing of 0.23 nm corresponds to the (111) plane of fcc Pt.

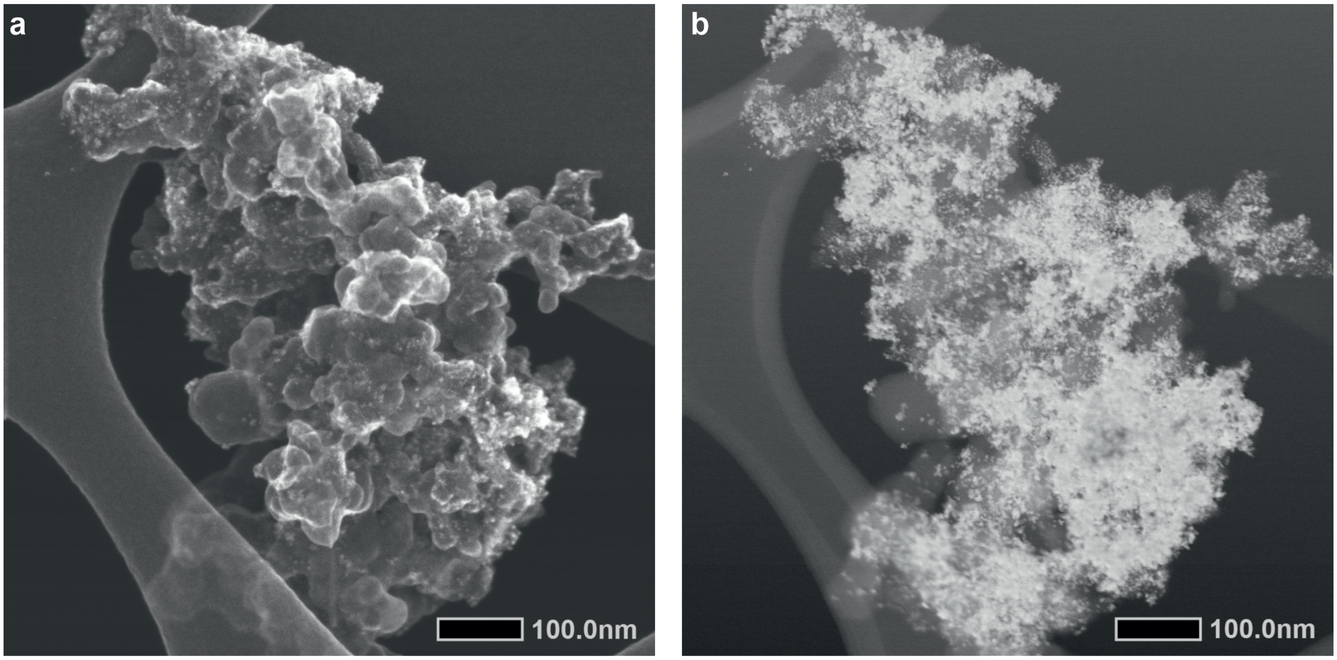

Figure S3. Scanning Transmission Electron Microscopy (STEM) image of the platinum catalyst and carbon support from a pristine MEA recorded with a (surface sensitive) secondary electron (SE) detector (a) and in High-Angle Annular Dark Field (HAADF) mode (b), respectively, at the same area as depicted in Figure S2a. The SE image reveals that the finely dispersed platinum nanoparticles (small white spots) are well separated on top of the carbon particles. The high density of platinum on top and bottom of the carbon particles gives rise to the high z-contrast of the HAADF image.

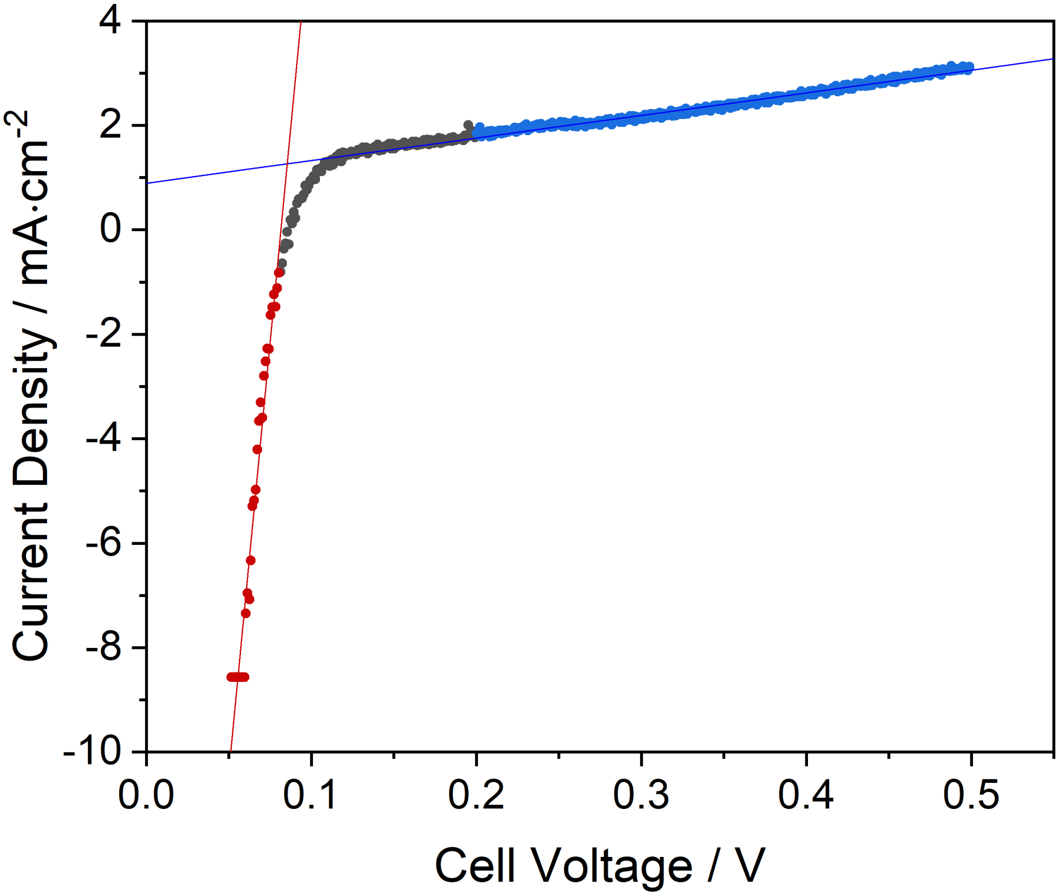

Figure S4. Examplary linear sweep voltammogram for determining the short-circuit resistance (reciprocal slope of blue line) and the hydrogen crossover current density (intersection between red and blue line).

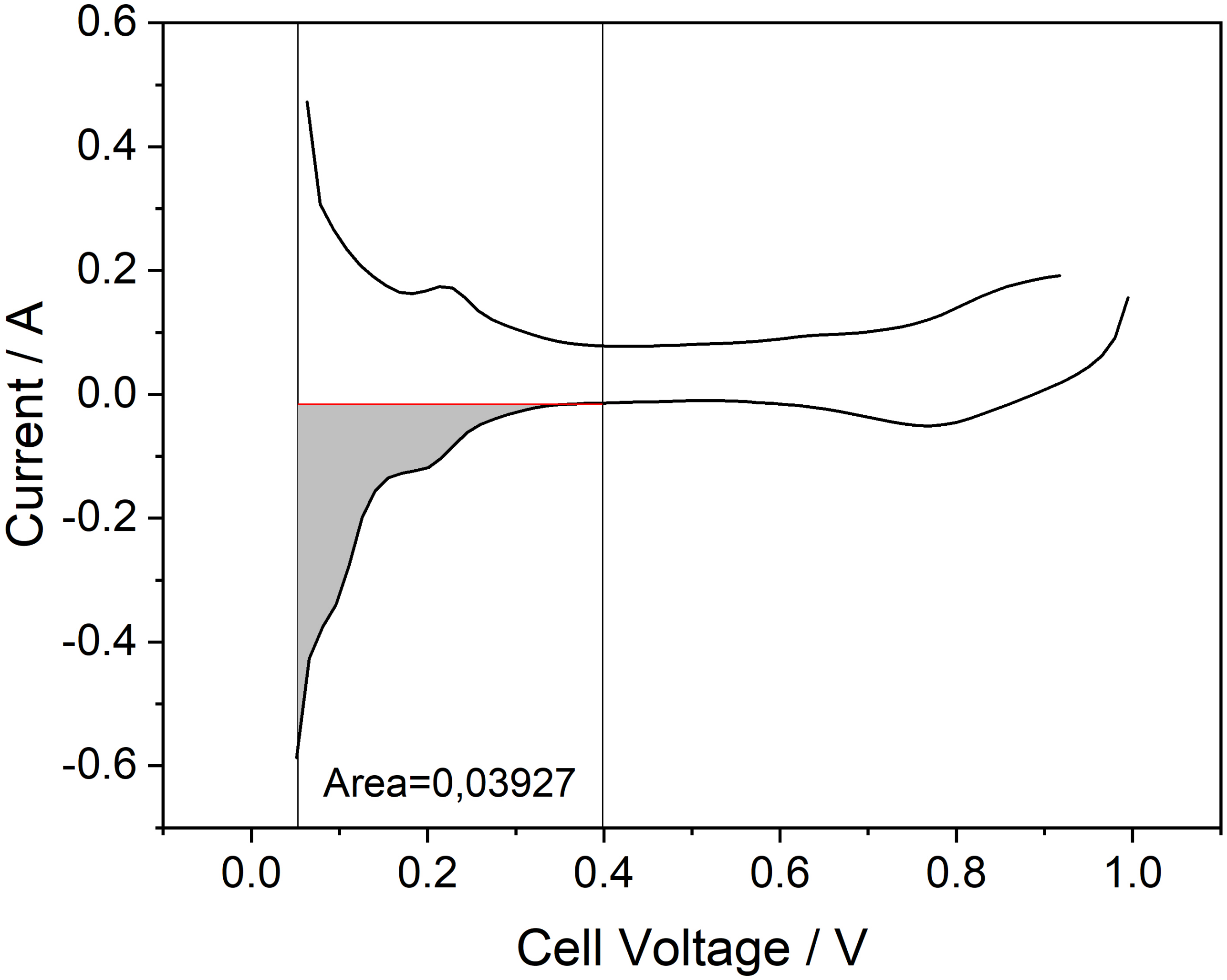

Figure S5. Exemplary cyclic voltammogram for the determination of the electrochemically active surface area (ECSA) by integration of the area under the peaks associated with hydrogen desorption (grey area).

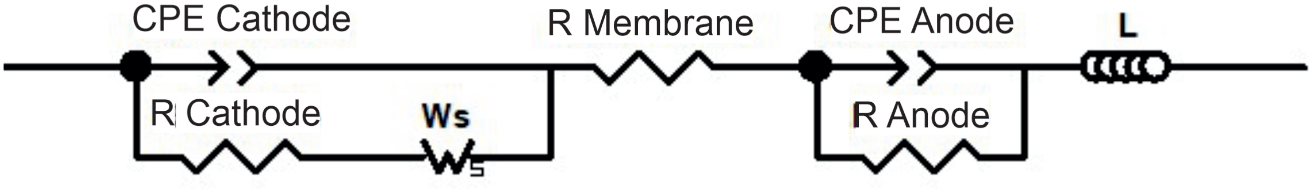

Figure S6. Equivalent circuit model used for the evaluation of the electrochemical impedance spectroscopy data.

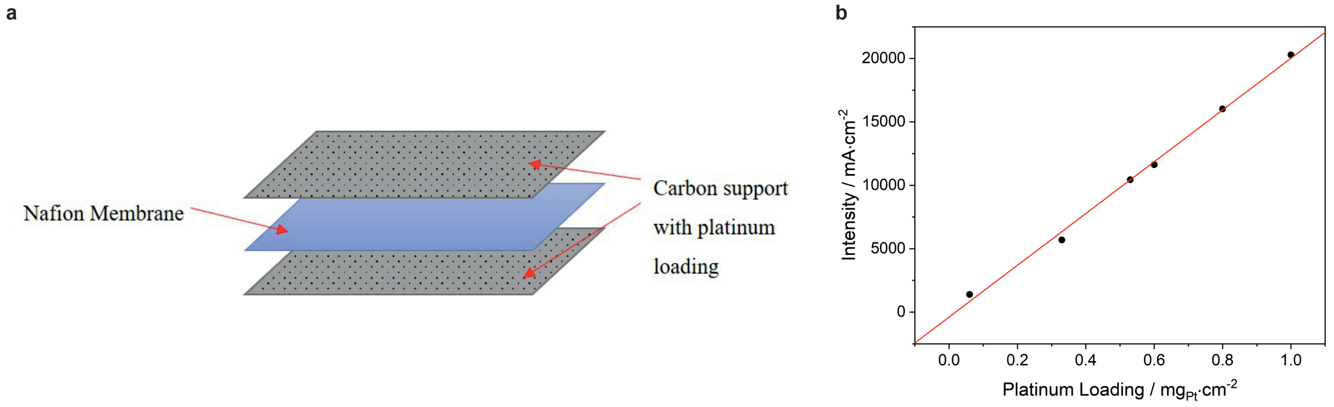

Figure S7. a) Measuring arrangement of membrane and carbon-supported catalyst layers to accommodate the XRF calibration line. b) Calibration line for the analysis of platinum in fuel cell MEAs by XRF.
